# LncRNA Sox2ot modulates the progression of thoracic aortic aneurysm by regulating miR-330-5p/Myh11

**DOI:** 10.1042/BSR20194040

**Published:** 2020-07-15

**Authors:** Weizhang Xiao, Xing Li, Cheng Ji, Jiahai Shi, Youmin Pan

**Affiliations:** 1 Department of Cardiothoracic Surgery, Affiliated Hospital of Nantong University, No.20 Xisi Road, Nantong 226001, Jiangsu, China; 2 Department of Cardiovascular Surgery, Tongji Hospital, Tongji Medical College of Huazhong University of Science and Technology, No.1095 Jiefang Avenue, Hankou 430030, Hubei, China

**Keywords:** miR-330-5p, Myh11, Sox2ot, thoracic aortic aneurysm

## Abstract

Thoracic aortic aneurysm (TAA) has been causing the death of elder people. Myosin heavy chain 11 (Myh11) has been reported associated with aortic aneurysm, but there is no specific study on its function on TAA. Here we aimed to explore the function of Myh11 on mouse aortic smooth muscle cells (SMCs) for studying the inner mechanism of TAA. H_2_O_2_ treatment was implemented on mouse aortic SMCs for detecting cell apoptosis. Meanwhile, functional assays were conducted to verify the function of Myh11 on mouse aortic SMCs. Also, pull-down assay, RIP assay were implemented to identify the potential RNAs for study. Quantitative real-time polymerase chain reaction (qRT-PCR) and luciferase reporter assay were implemented to identify the expression and binding relationships of RNAs. Myh11 expression was increased by treatment of H_2_O_2_. Myh11 could decrease proliferation and enhance apoptosis of mouse aortic SMCs. At the same time, mmu-miR-330-5p could bind to Myh11 and Sox2ot, forming a competing endogenous RNA (ceRNA) pathway to regulate the proliferation and apoptosis of mouse aortic SMCs. Moreover, both Sox2ot and Myh11 were proved to be up-regulated whereas miR-330-5p down-regulated in Fbn1^C1039G/+^ mice, the *in vivo* model of TAA. In a word, long noncoding RNA (lncRNA) Sox2ot modulates the progression of TAA by regulating miR-330-5p/Myh11 axis.

## Introduction

Thoracic aortic aneurysm (TAA) has been affecting people’s health and is considered as a serious vascular disease [1]. TAA mainly occurs after the age of 60, and male patients occupy 80% of TAA cases [2,3]. Most of the TAA cases are caused by the deterioration of atherosclerosis, and a few cases are caused by syphilis [3,4]. Meanwhile, surgical treatment has become the most effective therapy for TAA. However, the surgical procedure is complicated and has high risk [3–5]. Thence, it is of vital importance for us to explore the deeper molecular mechanisms in TAA.

Myosin heavy chain 11 (Myh11) has been reportedly connected to plenty of human diseases, such as gastric cancer, colorectal cancer and acute myeloid leukemia [6–8]. For instance, lowly expressed MYH11 is connected to the poor prognosis of colorectal cancer [8]. Meanwhile, MYH11 has been reported to participate in the progression of aortic aneurysm. For example, MYH11 is involved in the trans-differentiation of fibroblasts in aortic aneurysms [9]. MYH11 exerts its significant influence on the hereditary TAA [10,11]. However, the upstream mechanism of MYH11 has not been specifically scrutinized in TAA. In our study, we aimed to search the function of Myh11 in TAA and further explore its molecular mechanism.

Long noncoding RNAs (lncRNAs) have been studied as a group of RNAs with more than 200 nucleotides at length, and lacking the function of coding proteins [12]. Much evidence showed that lncRNAs play significant roles and have complicated mechanism in various human diseases, including TAA [13]. For example, PVT1 has been studied as an aortic aneurysm regulator by modulating the apoptosis of cells [13]. HIF1A-AS1 regulates the proliferation and apoptosis of vascular smooth muscle cells (SMCs), which might contribute to TAA pathogenesis [14]. LOXL1-AS expression is up-regulated in TAA patients, and regulates proliferation and apoptosis of aortic SMCs through up-regulating Giver [15].

Moreover, it was widely reported that lncRNAs serve as the competing endogenous RNA (ceRNA) in modulation of cellular processes in various diseases, including aortic aneurysm. The ceRNA refers to lncRNAs that endogenously compete with messenger RNAs (mRNAs) to bind with microRNAs (miRNAs), thus antagonizing the suppression of miRNAs on mRNAs. LINC00473 serves as a ceRNA against miR-212-5p to up-regulate BASP1, thus suppressing vascular SMCs viability to promote aneurysm formation [16]. NEAT1 functions as a ceRNA to facilitate formation of abdominal aortic aneurysm by elevating TULP3 [17]. GAS5 acts as a miR-21 sponge to release PTEN, and contributes to aortic aneurysm formation through facilitating SMCs apoptosis [18]. In our study, we searched out Sox2ot, which is connected to the molecular mechanism of Myh11 in TAA, and further scrutinized the function of Sox2ot in TAA.

## Materials and methods

### Animal studies

We conducted the animal studies following the guidelines of the Animal Care and Use Committee of Affiliated Hospital of Nantong University. Marfan (Fbn1^C1039G/+^) mice and WT mice were commercially obtained from were obtained from Jackson Laboratories (B6.129-Fbn1^tm1Hcd/J^, #01885). Six months later, peripheral blood samples were collected from inner canthus of both WT and Fbn1^C1039G/+^ mice without any anesthetization. After that, mice were killed via cervical dislocation. For plasma separation, samples were centrifuged at 1500×***g*** for 15 min. Then, samples were frozen at −80°C for RNA extraction. The assays were repeated independently thrice. The animal experiments took place in specific pathogen-free (SPF) environment of the laboratory in Affiliated Hospital of Nantong University. Besides, the animal study was approved by Ethics Committee of Affiliated Hospital of Nantong University.

### Cell culture and treatment

MOVAS and A10 (ATCC; Rockville, Maryland), the mouse aortic vascular SMCs, were available and grown in high-glucose DMEM (Gibco, Thermo Fisher Scientific, Inc., Waltham, MA). Cells were cultivated with 1% antibiotics and 10% fetal bovine serum (FBS; GIBCO) in humidified incubator with 5% CO_2_ at 37°C. MOVAS and A10 cells were treated with 800 μmol/l of H_2_O_2_ for 0, 8, 16, 24 h for detecting the apoptosis situation.

### Quantitative real-time polymerase chain reaction

One milliliter of TRIzol reagent (Invitrogen, Carlsbad, CA) was first added into the cell culture medium to extract the total RNAs. Reverse transcription was then performed by the PrimeScript RT reagent kit (Takara Bio, Inc., Otsu, Japan) to synthesize cDNA. PCR system was prepared with SYBR Green PCR Master Mix (Takara), with Glyceraldehyde-3 phosphate dehydrogenase (GAPDH) or U6 as endogenous control. All data normalization was conducted on the 2^−ΔΔ*C*_T_^ method. The assays were repeated independently thrice.

### Transfection plasmids

The designed short hairpin RNAs (shRNAs) and nonspecific shRNAs were available from Genepharma Company (Shanghai, China) to silence Myh11 and Sox2ot using transfection kit Lipofectamine 6000 (Invitrogen). Besides, the miR-330-5p mimics/inhibitor and negative control (NC) mimics/inhibitor were either procured from Genepharma Company for the overexpression or silencing of miR-330-5p. The pcDNA3.1-Myh11 and pcDNA3.1-NC (Genepharma) were acquired for overexpressing Myh11. Following 48 h of transfection, cells were reaped.

### Immunofluorescence staining

Cells of MOVAS and A10 on glass coverslips were fixed in 4% formaldehyde, permeabilized in 0.5% Triton X-100, then blocked in 3% bovine serum albumin. Thirty minutes later, anti-Ki-67 antibody (Abcam, Cambridge, MA) and secondary antibody (Abcam) were used. Following DAPI staining for nuclear detection, images were taken under confocal microscopy (Leica, Wetzlar, Germany). The assays were repeated independently thrice.

### Colony formation

Cells of MOVAS and A10 in six-well plates were seeded for 14-day culture, subsequently treated with 4% formaldehyde and 0.1% Crystal Violet in turn. Colony numbers were manually counted. The assays were repeated independently thrice.

### Terminal deoxynucleotidyl transferase-mediated dUTP-biotin nick end labeling (Tunel) staining

The processed cells of MOVAS and A10 were permeabilized for 5 min after fixing in 4% formaldehyde for 1 h. TUNEL-method was performed as per the user guide of *In Situ* Cell Death Detection Kit (Roche, Basel, Switzerland). After 4',6-Diamidino-2-Phenylindole, Dihydrochloride (DAPI) staining, TUNEL-positive cells were processed by fluorescence microscope. The assays were repeated independently thrice.

### Flow cytometer apoptosis assay

Apoptotic cells were also detected by Annexin V-FITC Apoptosis Detection Kit (BD Bioscience, San Jose, CA). Annexin V-FITC (AV) and propidium iodide (PI) were added to cell culture medium in line with the relative instructions. Results were all assayed by flow cytometry using FACS Calibur (BD Bioscience). The assays were repeated independently thrice.

### RNA pull down

Cell lysates from RIPA lysis buffer were employed to cultivate with the Myh11 biotin probe or control probe. Streptavidin agarose beads were subsequently added and cultured for 1 h. The relative miRNA enrichment was measured using quantitative real-time polymerase chain reaction (qRT-PCR). The assays were repeated independently thrice.

### Luciferase reporter assay

The fragments of Myh11 or Sox2ot covering the wild-type and mutated miR-330-5p binding sites were cloned into the pmirGLO vector, termed Myh11-WT/Mut and Sox2ot-WT/Mut. Followed by co-transfection with miR-330-5p mimics or NC-mimics, cells were collected after 48 h for processing with Luciferase Reporter Assay System (Promega, Madison, WI). The assays were repeated independently thrice.

### RNA immunoprecipitation

Cultured cells of MOVAS and A10 in PBS were trypsinized, cell lysates were acquired by centrifugation after incubation in RNA immunoprecipitation (RIP) lysis buffer. The beads–antibody mixture was prepared for culturing with cell lysates. After RNA extraction and purification, qRT-PCR was followed. The used antibodies against human protein argonaute-2 (Ago2) and control IgG were both available from Abcam. The assays were repeated independently thrice.

### Statistical analysis

All experiments in the present study were repeated independently thrice. Data were presented as the means ± standard deviations (S.D.). Significance of difference between groups was assayed by Student’s *t* test or one-way ANOVA followed by Tukey’s post-hoc test using GraphPad Prism V5.01. Threshold of *P*-value was defined statistically as 0.05.

## Results

### Inhibited Myh11 could increase cell proliferation and inhibit cell apoptosis in TAA

**Figure 1:**
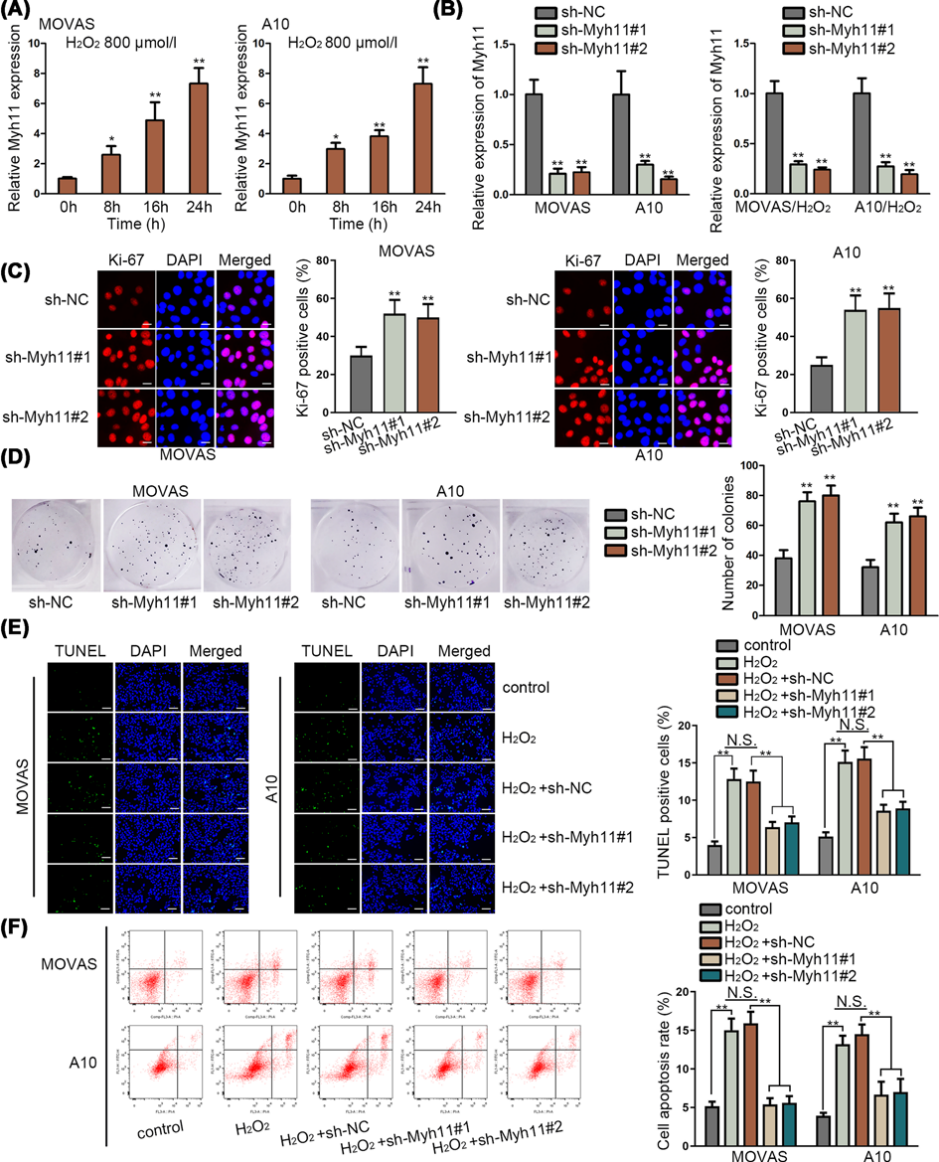
Inhibited Myh11 could increase cell proliferation and inhibit cell apoptosis in TAA (**A**) Expression of Myh11 was assessed by qRT-PCR assay in mouse aortic SMCs (MOVAS and A10), which were treated with H_2_O_2_ (800 μmol/l) at 0, 8, 16, 24 h. (**B**) Inhibition efficiency of Myh11 was detected via qRT-PCR assay in untreated MOVAS and A10 cells, and in H_2_O_2_ (800 μmol/l) treated MOVAS and A10 cells for 24 h. (**C,D**) Immunofluorescence assay (scale bar = 20 μm) and colony formation assay investigated the cell proliferation of MOVAS and A10 cells when Myh11 was inhibited. (**E,F**) TUNEL assay (scale bar = 200 μm) and flow cytometry analysis searched the apoptosis situation of MOVAS and A10 cells treated with H_2_O_2_ (800 μmol/l) for 24 h. *P<0.05, **P<0.01.

MYH11 made its significant influence on the hereditary TAA [10,11]. We studied the expression of Myh11 in mouse aortic SMCs (MOVAS and A10) at first. qRT-PCR assay revealed that Myh11 was up-regulated by H_2_O_2_ (800 μmol/l) treatment in a time-dependent manner (Figure 1A). Further, we detected the inhibition efficiency of Myh11 via qRT-PCR assay. The results depicted that Myh11 expression was down-regulated by transfection of sh-Myh11#1/2 to MOVAS and A10 cells with or without H_2_O_2_ treatment (Figure 1B). Furthermore, we implemented functional assays to identify the cell proliferation and apoptosis in MOVAS and A10 cells when Myh11 was down-regulated. Immunofluorescence assay and colony formation assay revealed that Ki-67 positive cells and number of colonies were both enhanced by silenced Myh11 in MOVAS and A10 cells (Figure 1C,D). TUNEL assay and flow cytometry analysis showed that increased cell apoptosis caused by H_2_O_2_ (800 μmol/l) treatment was significantly decreased again by inhibited Myh11 (Figure 1E,F). In a word, inhibited Myh11 could increase cell proliferation and inhibit cell apoptosis in mouse aortic SMCs.

### MiR-330-5p could bind to Myh11 in MOVAS and A10 cells

**Figure 2:**
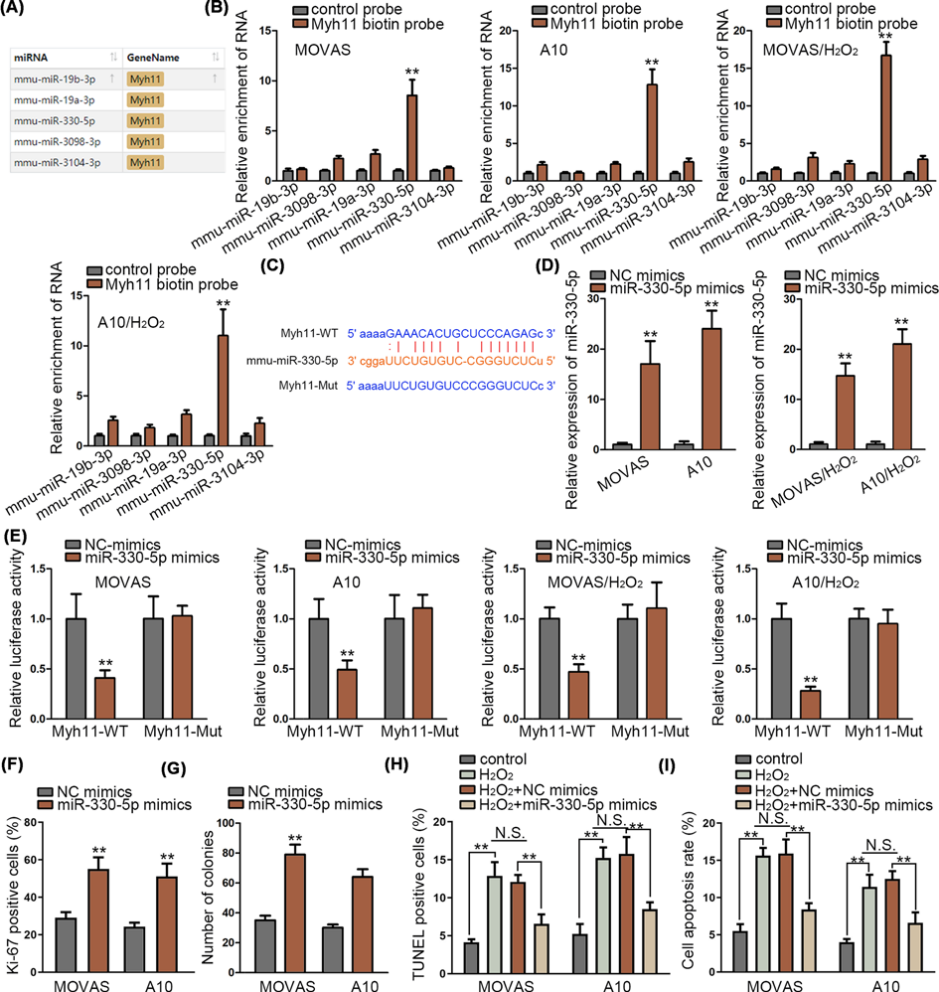
MiR-330-5p could bind to Myh11 in MOVAS and A10 cells (**A**) Five miRNAs were identified binding to Myh11 according to ENCORI (http://starbase.sysu.edu.cn/index.php). (**B**) RNA pull-down assay identified the binding of these five RNAs to Myh11 in untreated MOVAS and A10 cells, and in H_2_O_2_ (800 μmol/l) treated MOVAS and A10 cells (for 24 h). (**C**) The binding sites between mmu-miR-330-5p and Myh11 were presented by ENCORI prediction. (**D**) Overexpression efficiency of miR-330-5p was detected via qRT-PCR assay in untreated MOVAS and A10 cells, and in H_2_O_2_ (800 μmol/l) treated MOVAS and A10 cells (for 24 h). (**E**) Luciferase reporter assay tested the binding in miR-330-5p and Myh11 in untreated MOVAS and A10 cells, and in H_2_O_2_ (800 μmol/l) treated MOVAS and A10 cells (for 24 h). (**F**–**I**) Immunofluorescence assay, colony formation assay evaluated cell proliferation in MOVAS and A10 cells; TUNEL assay and flow cytometry analysis were implemented to study the cell apoptosis of MOVAS and A10 cells treated with H_2_O_2_ (800 μmol/l) for 24 h when miR-330-5p was overexpressed. **P<0.01.

Next, we identified the miRNAs which could bind to Myh11. ENCORI website was implemented to predict potential miRNAs which could bind to Myh11 (Figure 2A). Five miRNAs were searched out which were: mmu-miR-19b-3p, mmu-miR-3098-3p, mmu-miR-19a-3p, mmu-miR-330-5p and mmu-miR-3104-3p. Meanwhile, RNA pull-down assay was used to assess the binding of these five RNAs to Myh11. The results depicted that only mmu-miR-330-5p (named as miR-330-5p in other part of the manuscript) was significantly pulled down by biotin-labeled Myh11 (Figure 2B). The binding site of miR-330-5p and Myh11 was presented by ENCORI, and we mutated the binding sites of Myh11 (Figure 2C). Also, overexpression efficiency of miR-330-5p was verified via qRT-PCR assay (Figure 2D). Then, luciferase reporter assay was used to test the binding in miR-330-5p and Myh11. Results showed that luciferase activity of Myh11 wild-type was significantly decreased by miR-330-5p overexpression, while no changes were observed on luciferase activity of Myh11 mutant type (Figure 2E). Meanwhile, immunofluorescence assay and colony formation assay revealed that gain function of miR-330-5p elevated the proliferation of MOVAS and A10 cells (Figure 2F,G). Overexpressed miR-330-5p reduced the cell apoptosis in TUNEL assay and flow cytometry analysis (Figure 2H,I). In conclusion, miR-330-5p could bind to Myh11 and exert pro-proliferation and anti-apoptosis effects in MOVAS and A10 cells.

### Sox2ot could bind to miR-330-5p in MOVAS and A10 cells

**Figure 3:**
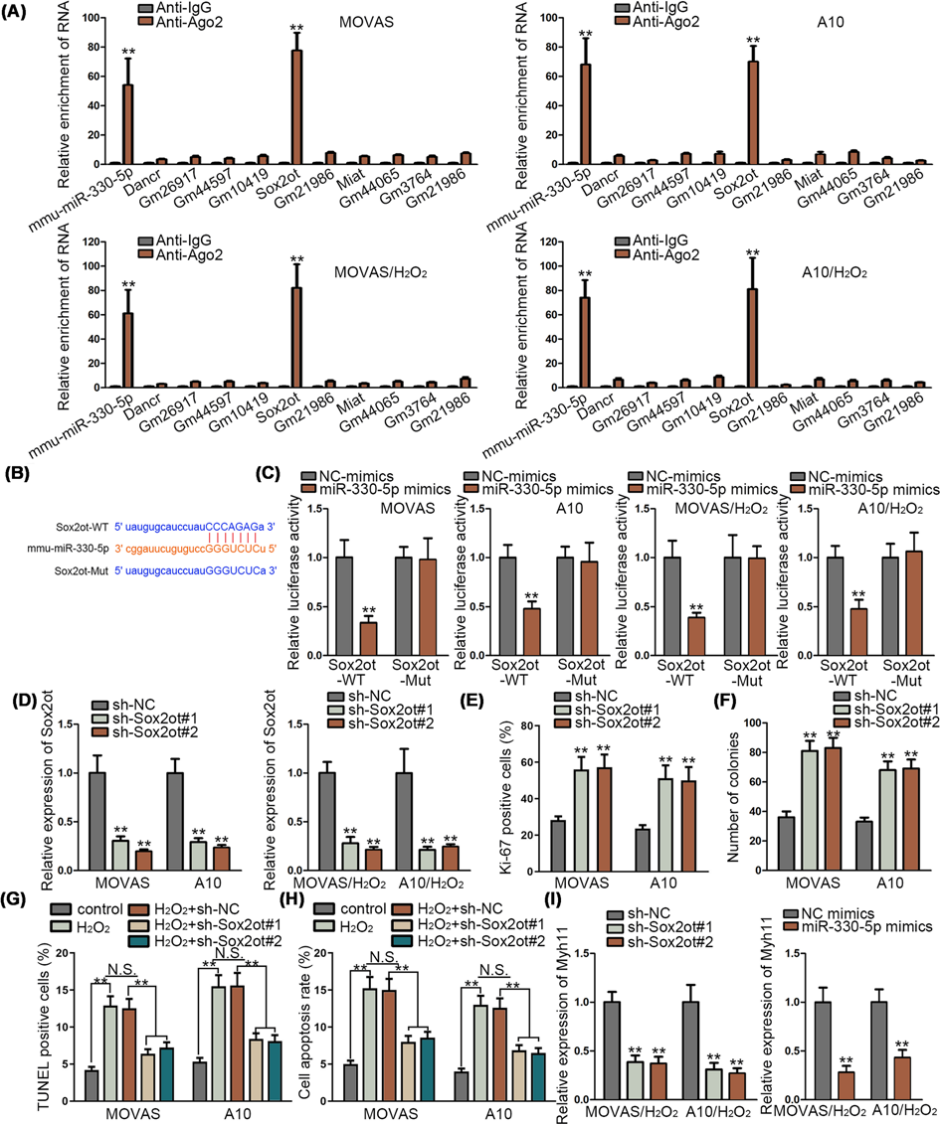
Sox2ot could bind to miR-330-5p in MOVAS and A10 cells (**A**) RIP assay was conducted to search the possible lncRNAs which could bind to miR-330-5p in untreated MOVAS and A10 cells, and in H_2_O_2_ (800 μmol/l) treated MOVAS and A10 cells (for 24 h). (**B**) The binding sites between miR-330-5p and Sox2ot were presented according to ENCORI. (**C**) Luciferase reporter assay was implemented to verify the binding in miR-330-5p and Sox2ot in untreated MOVAS and A10 cells, and in H_2_O_2_ (800 μmol/l) treated MOVAS and A10 cells (for 24 h). (**D**) Inhibition efficiency of Sox2ot was tested by qRT-PCR assay in untreated MOVAS and A10 cells, and in H_2_O_2_ (800 μmol/l) treated MOVAS and A10 cells (for 24 h). (**E**–**H**) Immunofluorescence assay, colony formation assay, TUNEL assay and flow cytometry analysis were implemented to study the cell proliferation and apoptosis of MOVAS and A10 cells when Sox2ot was silenced. For TUNEL assay and flow cytometry assay, cells were treated with H_2_O_2_ (800 μmol/l) for 24 h. (**I**) Expression of Myh11 was investigated via qRT-PCR assay in MOVAS and A10 cells treated with H_2_O_2_ (800 μmol/l) for 24 h when Sox2ot was silenced or miR-330-5p was overexpressed. **P<0.01.

LncRNAs are involved in the progression of TAA [19]. Subsequently, we planned to identify the lncRNA which could bind to miR-330-5p in MOVAS and A10 cells via RIP assay. Results showed that both mmu-miR-330-5p and Sox2ot were enriched in the anti-Ago2 in MOVAS and A10 cells, which indicated that miR-330-5p and Sox2ot had a connection in MOVAS and A10 cells (Figure 3A). Meanwhile, the binding site of miR-330-5p and Sox2ot was presented, as predicted by ENCORI; we mutated the binding sites of Sox2ot to examine whether the predicted sites were responsible for the interaction of miR-330-5p and Sox2ot (Figure 3B). Luciferase reporter assay was implemented to verify the binding in miR-330-5p and Sox2ot. We found that luciferase activity of Sox2ot wild type was decreased by overexpressed miR-330-5p (Figure 3C). Also, inhibition efficiency of Sox2ot was verified by qRT-PCR assay in MOVAS and A10 cells (Figure 3D). Meanwhile, Sox2ot has been reported connected to the ischemic heart failure [20]. Subsequently, the functional experiments were designed to evaluate the proliferation and apoptosis of MOVAS and A10 cells when Sox2ot was silenced. Immunofluorescence assay and colony formation assay delineated that cell proliferation was promoted by Sox2ot inhibition (Figure 3E,F). Also, cell apoptosis was decreased by depleted Sox2ot, as was detected by TUNEL assay and flow cytometry analysis (Figure 3G,H). Meanwhile, expression of Myh11 was investigated via qRT-PCR assay in MOVAS and A10 cells when Sox2ot was silenced or miR-330-5p was overexpressed. Myh11 expression was significantly inhibited by Sox2ot depletion and miR-330-5p overexpression (Figure 3I). In conclusion, Sox2ot could bind to miR-330-5p, and inhibited Sox2ot could increase cell proliferation and inhibit cell apoptosis in TAA.

### Sox2ot/miR-330-5p/Myh11 axis could modulate TAA progression

**Figure 4:**
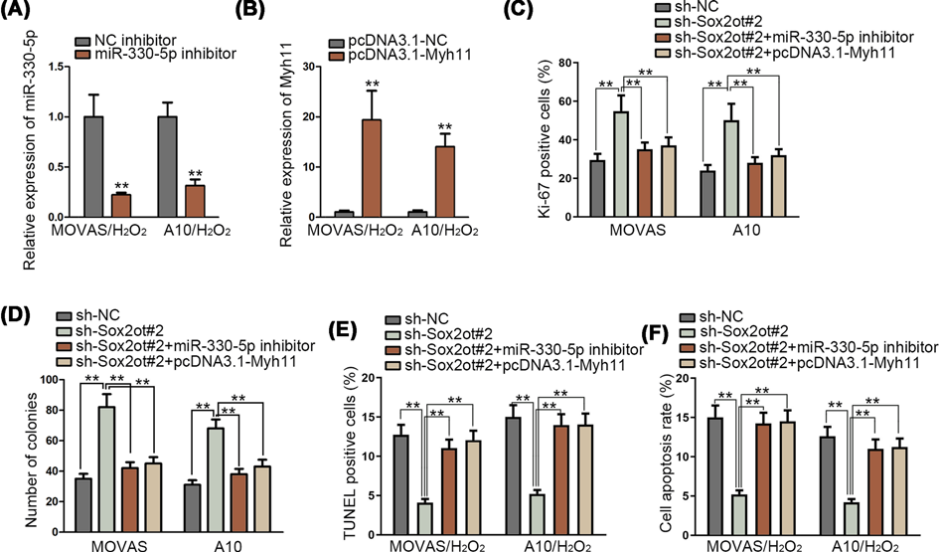
Sox2ot/miR-330-5p/Myh11 axis could modulate the progression of TAA (**A,B**) Inhibition efficiency of miR-330-5p and overexpression efficiency of Myh11 was verified via qRT-PCR assay in MOVAS and A10 cells treated with H_2_O_2_ (800 μmol/l) for 24 h. (**C**–**F**) Rescue experiments of immunofluorescence assay, colony formation assay, TUNEL assay and flow cytometry analysis investigated the cell proliferation and apoptosis of MOVAS and A10 cells. For TUNEL assay and flow cytometry assay, cells were treated with H_2_O_2_ (800 μmol/l) for 24 h. **P<0.01.

We further tested the influence of Sox2ot/miR-330-5p/Myh11 interaction on the TAA. First of all, qRT-PCR assay validated the inhibition efficiency of miR-330-5p and overexpression efficiency of Myh11 (Figure 4A,B). Meanwhile, rescue assays were made in *in vitro* model of TAA, MOVAS and A10 cells. We divided assays into four groups: sh-NC, sh-Sox2ot#1, sh-Sox2ot+miR-330-5p inhibitor, sh-Sox2ot+pcDNA3.1-Myh11. Immunofluorescence assay and colony formation assay demonstrated that inhibited Sox2ot could increase the cell proliferation, while such effect was restored by silenced miR-330-5p and Myh11 overexpression (Figure 4C,D). Also, MOVAS and A10 cells were treated with H_2_O_2_ (800 μmol/l) to induce apoptosis. Decreased cell apoptosis, which was caused by Sox2ot inhibition, was elevated again by silenced miR-330-5p and Myh11 overexpression in TUNEL assay and flow cytometry analysis (Figure 4E,F). In brief, Sox2ot/miR-330-5p/Myh11 axis could modulate cell proliferation and apoptosis of MOVAS and A10 cells.

### Sox2ot/Myh11/miR-330-5p axis was involved in TAA progression in mice

**Figure 5:**
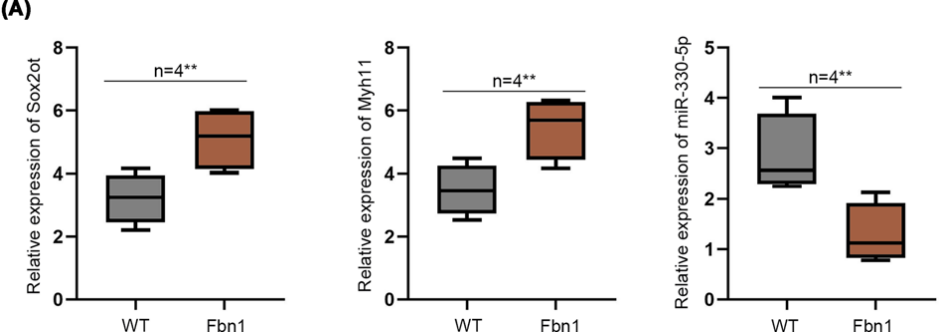
Sox2ot/Myh11/miR-330-5p axis was involved in TAA progression in mice (**A**) Relative expression of Sox2ot, Myh11, miR-330-5p in the plasma of Fbn1^C1039G/+^ and WT mice was verified by qRT-PCR assay. **P<0.01.

Then, mice model of TAA was applied to examine Sox2ot/Myh11/miR-330-5p axis *in vivo*. We detected expression of Sox2ot, Myh11, miR-330-5p in the plasma of Fbn1^C1039G/+^ mice compared with WT mice. The results depicted that Sox2ot and Myh11 were significantly up-regulated in the plasma of Fbn1^C1039G/+^ mice compared with WT mice. However, miR-330-5p was significantly down-regulated in the plasma of Fbn1^C1039G/+^ mice (Figure 5A). In summary, Sox2ot/Myh11/miR-330-5p axis was involved in TAA progression in mice.

## Discussion

TAA is a serious vascular disease and can lead to death. There is no effective medicine for treating TAA, and most of TAA patients were cured by operation surgery. Meanwhile, the aberrant proliferation and apoptosis of aortic SMCs have been identified as the characters of TAA [21–23]. Thence, we searched the biological behavior of cell proliferation and apoptosis of mouse aortic SMCs in TAA.

mRNA has been reported to participate in the progression of TAA. For instance, Talin-1 meditates the cell growth and migration in aortic dissection [22]. Meanwhile, BRG1 is up-regulated in TAA, and modulates the cell growth and apoptosis in TAA [23]. Also, MYH11 has been reported to participate in the progression of TAA [10,24]. In our study, we scrutinized the function of Myh11 in mouse aortic SMCs. qRT-PCR assay was conducted to test the expression of Myh11 in mouse aortic SMCs, and we revealed that H_2_O_2_ (800 μmol/l) treatment increased the expression of Myh11. Meanwhile, immunofluorescence assay and colony formation assay depicted the cell proliferation. TUNEL assay and flow cytometry analysis detected the cell apoptosis. We revealed that inhibited Myh11 significantly increased cell proliferation and decreased cell apoptosis of the mouse aortic SMCs.

LncRNAs are involved in the progression of TAA. For example, lncRNA HIF1A-AS1A can suppress cell apoptosis in TAA [19]. Also, HOTAIR expression is inhibited in TAA specimen, and inhibited HOTAIR promotes the cell apoptosis and suppresses the cell growth in TAA [25]. LncRNA MIAT has been identified to enhance the progression of TAA by regulating the expression of miRNA-145 and modulating the PI3K/Akt pathway [26]. In our study, we searched the function of Sox2ot. In previous study, Sox2ot has been reported to meditate the progression of glioblastoma stem cells by regulating the miR-194-5p and miR-122 expression [27]. Sox2ot can enhance epithelial–mesenchymal transition (EMT) progression in pancreatic ductal adenocarcinoma by modulating other RNA expression [28]. Meanwhile, Sox2ot has been reported connected to the ischemic heart failure [20]. In our study, we first identified the miRNAs which could bind to both Myh11 and Sox2ot. RNA pull-down assay, luciferase reporter assay and RIP assay showed that miR-330-5p could bind to Myh11 and Sox2ot. Also, functional assays revealed that inhibited Sox2ot can increase proliferation and decrease apoptosis in mouse aortic SMCs. Moreover, Sox2ot and Myh11 were significantly up-regulated in the plasma of TAA mice, while miR-330-5p was significantly down-regulated in the plasma of TAA mice. In a word, lncRNA Sox2ot could modulate the progression of TAA by regulating miR-330-5p/Myh11 axis.

## Abbreviations

Ago2: protein argonaute-2
ceRNA: competing endogenous RNA
DAPI: 4′,6-Diamidino-2-Phenylindole, Dihydrochloride
EMT: epithelial–mesenchymal transition
GAPDH: glyceraldehyde-3 phosphate dehydrogenase
lncRNA: long noncoding RNA
miRNA: microRNA
mRNA: messenger RNA
Myh11: myosin heavy chain 11
NC: negative control
qRT-PCR: quantitative real-time polymerase chain reaction
RIP: RNA immunoprecipitation
shRNA: short hairpin RNA
SMC: smooth muscle cell
TAA: thoracic aortic aneurysm
Tunel: terminal deoxynucleotidyl transferase-mediated dUTP-biotin nick end labeling assay
